# Human papillomavirus in the setting of immunodeficiency: Pathogenesis and the emergence of next-generation therapies to reduce the high associated cancer risk

**DOI:** 10.3389/fimmu.2023.1112513

**Published:** 2023-03-07

**Authors:** Rehana V. Hewavisenti, Joshua Arena, Chantelle L. Ahlenstiel, Sarah C. Sasson

**Affiliations:** ^1^ Immunovirology and Pathogenesis Program, The Kirby Institute, The University of New South Wales, Sydney, NSW, Australia; ^2^ UNSW RNA Institute, The University of New South Wales, Sydney, NSW, Australia

**Keywords:** human papillomavirus (HPV), human immunodeficiency virus (HIV), RNA interference, squamous cell carcinoma, transplant, primary immunodefciencies, cancer, nanoparticles

## Abstract

Human papillomavirus (HPV), a common sexually transmitted virus infecting mucosal or cutaneous stratified epithelia, is implicated in the rising of associated cancers worldwide. While HPV infection can be cleared by an adequate immune response, immunocompromised individuals can develop persistent, treatment-refractory, and progressive disease. Primary immunodeficiencies (PIDs) associated with HPV-related disease include inborn errors of GATA, EVER1/2, and CXCR4 mutations, resulting in defective cellular function. People living with secondary immunodeficiency (e.g. solid-organ transplants recipients of immunosuppression) and acquired immunodeficiency (e.g. concurrent human immunodeficiency virus (HIV) infection) are also at significant risk of HPV-related disease. Immunocompromised people are highly susceptible to the development of cutaneous and mucosal warts, and cervical, anogenital and oropharyngeal carcinomas. The specific mechanisms underlying high-risk HPV-driven cancer development in immunocompromised hosts are not well understood. Current treatments for HPV-related cancers include surgery with adjuvant chemotherapy and/or radiotherapy, with clinical trials underway to investigate the use of anti-PD-1 therapy. In the setting of HIV co-infection, persistent high-grade anal intraepithelial neoplasia can occur despite suppressive antiretroviral therapy, resulting in an ongoing risk for transformation to overt malignancy. Although therapeutic vaccines against HPV are under development, the efficacy of these in the setting of PID, secondary- or acquired- immunodeficiencies remains unclear. RNA-based therapeutic targeting of the HPV genome or mRNA transcript has become a promising next-generation therapeutic avenue. In this review, we summarise the current understanding of HPV pathogenesis, immune evasion, and malignant transformation, with a focus on key PIDs, secondary immunodeficiencies, and HIV infection. Current management and vaccine regimes are outlined in relation to HPV-driven cancer, and specifically, the need for more effective therapeutic strategies for immunocompromised hosts. The recent advances in RNA-based gene targeting including CRISPR and short interfering RNA (siRNA), and the potential application to HPV infection are of great interest. An increased understanding of both the dysregulated immune responses in immunocompromised hosts and of viral persistence is essential for the design of next-generation therapies to eliminate HPV persistence and cancer development in the most at-risk populations.

## 1 Introduction

Approximately 15-20% of global cancers are associated with oncogenic viral infections (1, 2). These viruses adopt numerous mechanisms by which they evade immune responses and establish persistent infections resulting in malignant transformation (3). A common trait among oncogenic viruses is that only a small proportion of chronically-infected cells develop cancer (4). Viruses such as Human Papillomavirus (HPV), Epstein-Barr Virus (EBV), Hepatitis B virus (HBV), and Hepatitis C virus (HCV) are known to be implicated in the pathogenesis of human cancer (5). These viruses act as obligate intracellular parasites, where the microbe encodes proteins that affect cell development, apoptosis, and growth cycle. The virus ‘reprograms’ host cellular signaling, disrupting major checkpoints regulating proliferation, differentiation and cell death, and genomic integrity (3). Immunosurveillance, important in identifying and removing aberrant cells from the proliferative pool, is also negatively impacted (3). Additionally, cancer progression as a result of chronic viral infection is dependent on host factors, including local and systemic immunity, somatic mutations, immunosuppression, genetic predisposition, and environmental factors, such as carcinogen exposure.

HPV is a double-stranded DNA virus associated with cancers of the squamous epithelia of the cervix, oropharynx, and anogenital regions. There are currently ~200 identified HPV types, with many capable of causing a range of mucosal or cutaneous epithelial hyperplastic lesions (6). These types are divided into low-risk HPV (LR-HPV) and high-risk HPV (HR-HPV) groups depending on their likelihood of malignant progression (7, 8). Immunosuppressed individuals are at a heightened risk of developing HPV-associated lesions and cancers, making HPV a useful model for understanding viral-host interactions leading to epithelial-derived tumors. The most well-understood HPV-induced cancer is cervical cancer, with 99% being related to HR-HPV types (3). However, HPV-associated cancer affects many other mucosal sites, with 64–91% of vaginal, 40–50% of vulvar, 88–94% of anal, and 40–50% of penile cancers being HPV-associated (9).

Immunosurveillance plays a crucial role in initiating antigen-dependent responses in eliminating HPV infection and virally transformed cells. In immunocompetent hosts, 90% of anogenital HPV infections are cleared (10, 11). Innate immune cells such as natural killer (NK) cells, dendritic cells (DCs) and Langerhans cells (LCs) play an important role during the initial onset of infection, while HPV-specific CD8^+^ T cells target early viral proteins in infected cells (9, 12, 13). Conversely, HPV-associated malignancies have been commonly associated with at-risk population groups, including organ transplant recipients, systemic immunosuppressed patients, and people living with HIV (PLWH). Although HPV can evade immunity for long periods of time in immunocompetent hosts, infection is generally resolved. However, HPV infection can be persistent in immunocompromised individuals, with extensive HPV disease manifesting as non-regressive high-grade intraepithelial lesions (HSILs), leaving these individuals susceptible to HPV-associated malignancy formation (14–18).

**Figure 1:**
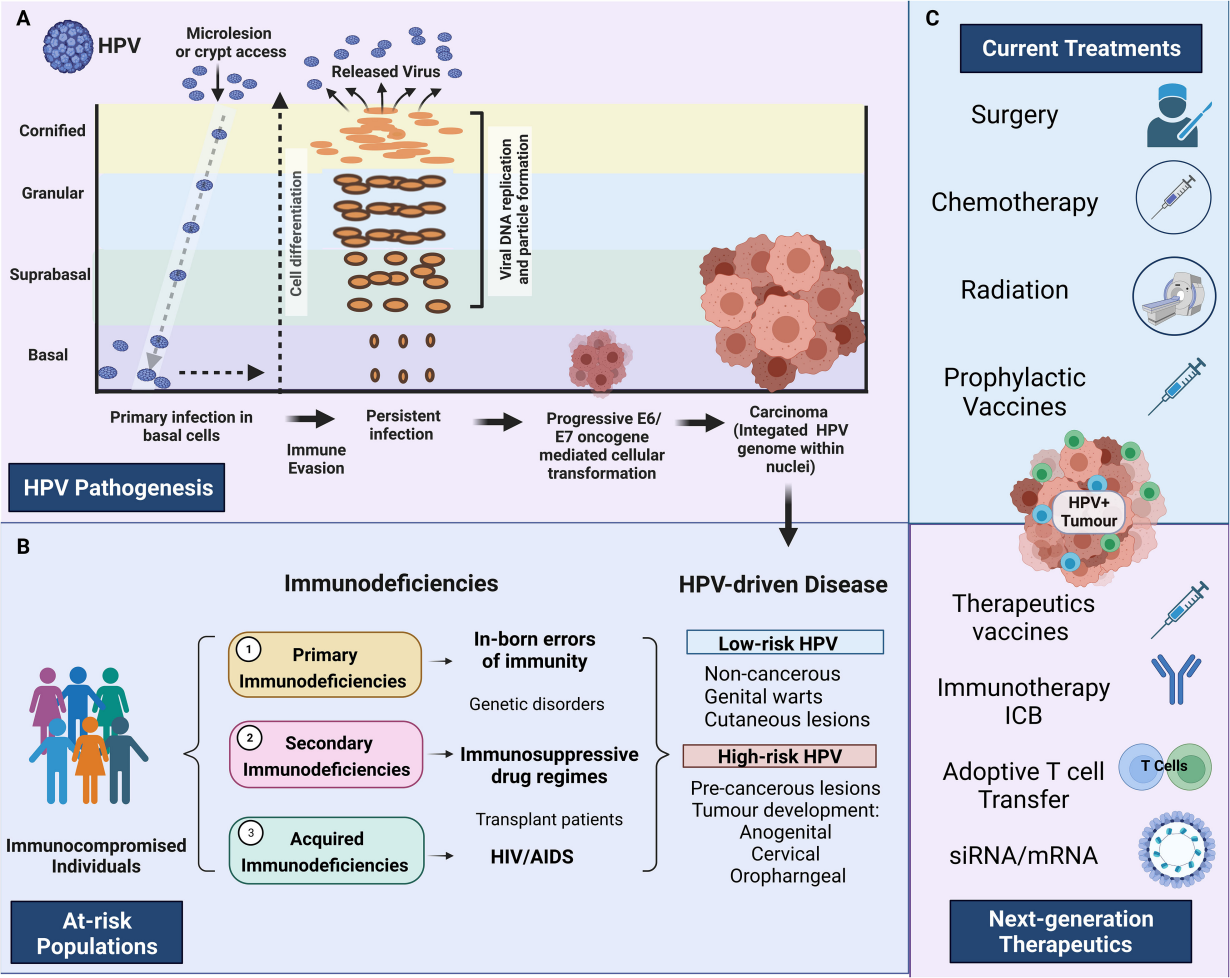
HPV pathogenesis and treatment in at-risk individuals. **(A)** HPV pathogenesis, persistent infection, and progression to cancer. Primary infection occurs when HPV gains access to basal cells through microlesions or damage to the skin. Upon viral replication, HPV can evade the immune response resulting in persistent disease. E6 and E7 oncogenes disrupt the cell cycle, which can result in persistent disease, cellular transformation and HPV-driven carcinoma. **(B)** Describes the three main immunodeficiencies 1) Primary immunodeficiencies relating to inborn errors of immunity, 2) Secondary immunodeficiencies relating to those under immunosuppressive drugs 3) Acquired immunodeficiencies relating to people living with HIV/AIDS. The major HPV pathologies caused by high-risk HPV and low-risk HPV strains are identified on the extreme right-hand side panel (HPV-driven disease) **(C)** Identifies the main treatments currently available and the next-generation therapeutics currently under investigation. Created with BioRender.com.

Current therapeutic regimens for the treatment of HPV-associated cancers can include surgery, chemotherapy and/or radiotherapy. HPV-associated cancers are treated medically the same as HPV cancers, despite different biological origins. This approach is often insufficient and can be associated with poor survival rates (19). Globally, the 5-year survival rate of cervical carcinomas is 64%, penile squamous cell carcinomas (SCCs) is 47%, anal SCCs is 70%, rectal SCCs is 56%, and oropharyngeal SCC is 51% (20). In immunocompromised individuals, these survival statistics are lower. HIV-positive (HIV+) women with cervical cancer have a 3-year survival of 35% (21) whilst those with anal squamous cell carcinoma (ASCC) have a 5-year survival rate of 47% (22). Effective treatments for immunocompromised individuals are challenging due to the limited understanding of the varying immune responses to disease, which are highly dependent on host factors. A greater understanding of these underlying immune mechanisms in at-risk individuals is crucial for the future design of next-generation therapeutics and technologies targeting HPV. The prevention of persistent HPV infection and cancerous progression in at-risk groups is a priority globally. Here, we discuss the current understanding of HPV pathogenesis, key challenges in current clinical management, and the emergence of next-generation RNA therapeutics, focusing on at-risk populations (**Figure 1**).

## 2 HPV epidemiology and risk factors

### 2.1 HPV epidemiology

**Figure 2:**
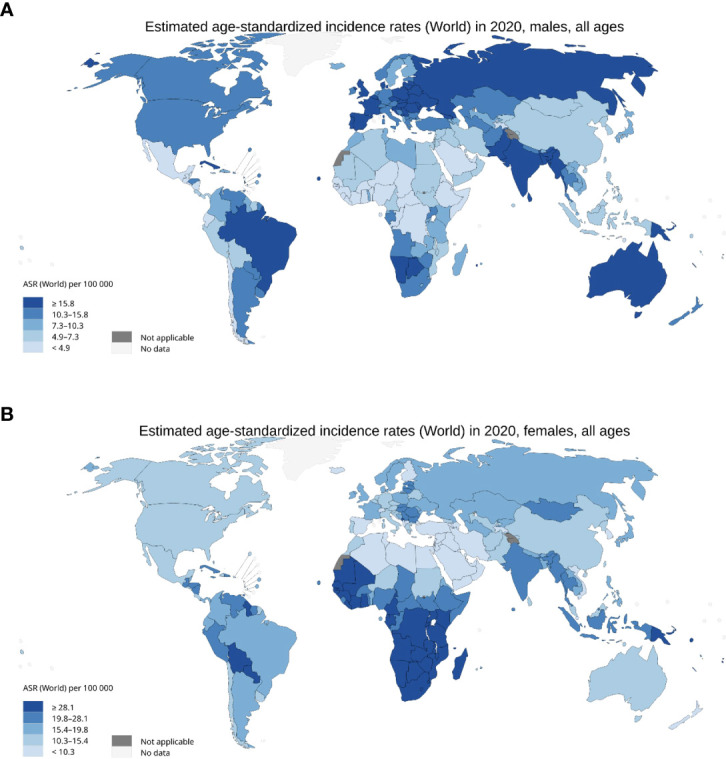
Incidence of HPV associated malignancies in 2020. **(A)** Age standardised rate per 100,000 for men with cancer of the anus, oropharyngeal, larynx, penis, lips, and oral cavity. **(B)** Age standardised rate per 100,000 for females with cancer of the anus, cervix uteri, larynx, oropharynx, lip, and oral cavity. International Agency for Research on Cancer, World Health Organisation 2020.

HPV is endemic globally, representing the most common sexually transmitted disease with 84% of sexually active women and 91% of sexually active men estimated to acquire the infection during their lifetime (23). HPV prevalence differs by age, with elevated rates of incidence amongst the 18-21 and 55–65 year-old cohorts. HPV is present in ~11.4% of the worldwide population, with the highest prevalence in Sub-Saharan Africa (24%), Eastern Europe (21%), and Latin America (16%) (24). In the period 1990-2012, the global incidence of HPV-associated malignancies reduced by 0.3%, but varied between geographic regions, with decreased incidence linked to strong HPV vaccination regiments in developed nations and increased incidence in countries with weaker healthcare infrastructure (25, 26) (**Figure 2**). Consequently, HPV infection is still implicated in the pathology of approximately 5% of all cancers (27).

### 2.2 Risk factors associated with HPV progression to cancerous lesions

HPV infection alone is not sufficient for the formation of HPV-associated cancers (28). Studies have aimed to identify the co-factors that predict and are associated with HPV infection progression to cancer, with major findings summarised below.

#### 2.2.1 HPV type

HPV type is the primary factor associated with infectious progression to cancerous lesions. HR-HPV is a group 1 carcinogen and includes types 16, 18, 31, 33, 35, 39, 45, 51, 52, 56, 58, 59, and 66 (29). HR-HPVs play a crucial role in the etiology and pathophysiology of malignancies of the cervix, anus, oropharynx, vulva, and penis (9, 30–34). LR-HPV genotypes include 6, 11, 40, 42, 43, 44, 54, 61, 70, 72, and 81, and can cause benign or low-grade cervical tissue changes and genital warts that seldom progress to cancerous lesions (35, 36). Of all the subtypes, only LR-HPVs 6 and 11 are classed as group 2B carcinogens (37).

#### 2.2.2 Tobacco smoking

Tobacco smoke is classed as a group A carcinogen by the World Health Organisation, containing >4500 chemical compounds, with at least 70 oncogenic to humans (38). Inhalation and use of tobacco-containing products have been significantly associated with the progression of HR-HPV infection to pre-cancerous and squamous cell carcinomas of the head and neck, cervix, anus, and penis (39–42). The precise mechanism by which tobacco smoke exacerbates the pathogenicity of HPV remains unknown, however recent studies suggest that tobacco comprises chemicals that enhance viral oncogenic expression in HPV-infected epithelium (43).

#### 2.2.3 High parity

High parity has been suspected of playing a role in the pathophysiology of HPV-associated carcinogenesis. A 2002 International Agency for Research on Cancer (IARC) multicentric case-control study found a direct positive association between the number of full-term pregnancies and the onset of HPV-associated squamous cell carcinoma, but not adenocarcinoma or adenosquamous carcinoma (44). Similarly, a 2022 meta-analysis of 6685 parous women found a 2.65 higher odds ratio for the development of cancerous lesions existed compared to their null parity counterparts (45). It is hypothesized that increased levels of progesterone and estrogen during the final weeks of pregnancy alter tight junctions between squamous and columnar epithelium, increasing the oncogenic activity of HPV (45).

#### 2.2.4 Oral hormonal contraception

The increased uptake of oral hormonal contraceptive use amongst women has renewed interest in the potential impact of associated HPV infection progression. There is some evidence suggesting an association between the long-term use of oral hormonal contraception with an increased risk in the development of HPV-associated cervical and anogenital carcinomas (46). However, a 2022 systematic review of 7 prospective studies did not find supporting evidence to suggest a significant association between oral contraceptive use and an increased risk of HPV-associated malignancies (47). Further investigation into the relationship between oral hormonal contraceptives and HPV disease progression is required.

#### 2.2.5 Diet

Dietary intake has been hypothesized to impact HPV clearance and resolution, though there remains no scientific consensus on the individual dietary components associated with resolution. Conflicting evidence surrounding the intake of vitamin A (48–50), B–Carotene (51–53) and folate (54–57) does not enable a determination as to their effect on the clinical resolution of HPV or cancerous lesion formation. Consequently, it remains uncertain as to the impact of diet in cases of HPV-associated cancers.

#### 2.2.6 Immunodeficiencies

Immunodeficiency is one of the highest risk factors associated with severe clinical manifestations of HPV-associated cancers. Immunodeficiencies are categorized into three main groups; 1) Primary immunodeficiencies (PIDs), 2) Secondary immunodeficiencies, and 3) Acquired immunodeficiency e.g. HIV infection. PIDs are inborn errors of immunity (58) and correlate with a higher prevalence of HPV-associated conditions when compared to healthy controls (59). Secondary immunodeficiencies include states of malnutrition and immunosuppression. Immunosuppression therapies include regimens prescribed to organ transplant recipients to reduce the risk of graft rejection. Over 30 tumor types have been evident at an increased frequency in individuals following transplantation, with the most common being SCCs (60). Acquired immunodeficiencies, predominately HIV infection, remain a significant co-factor in the pathophysiology of HPV-associated malignancies.

## 3 HPV biology and pathogenesis

### 3.1 HPV genome

HPVs are small, ~8 kb double-stranded DNA viruses that contain eight open reading frames (ORFs): E1, E2, E4, E5, E6, E7, L1 and L2 (61). E1, E2, E4 and E6 maintain viral function, whilst L1 and L2 encode structural capsid proteins required for viral replication (62). Two major promoters regulate transcription within the HPV genome: the early promoter located in the long control region, which regulates the expression of E6 and E7; and the late promoter located within the E7 gene, which regulates the expression of E1, E2, E4, E5, L1 and L2 (63).

HPVs are classed phylogenetically into five distinct genealogical classes with α-papillomaviruses uniquely constituting the HR-HPV and LR-HPV subtypes, as described above (64). HPV subtypes and variants are declared based on the genetic distance between viral genomes (65). A new HPV subtype is declared when the L1 ORF differs from any other known HPV type by ≥10%. Within types, variants exhibit between 0.5-1% genome differentiation (66). As of 2022, over 200 different HPV subtypes have been identified (67). Variation amongst HR-HPV subtypes poses a significant risk to the development of a singular therapeutic to target global prevalence (65).

### 3.2 HPV pathogenesis and immune evasion

HPV oncogenes can contribute to viral replication and cellular aberrations. E6 and E7 are the two primary oncogenes that promote HPV-associated carcinogenesis. HPV-associated cancers are more likely to occur in combination with the aforementioned risk factors including smoking, immunocompromise, or genomic alterations of host cell DNA (3). The HR-HPV types most associated with cancerous formation (in combination with host factors) are HPV16 and HPV18 (35, 68).

Factors that influence HPV infection and immune evasion include host environmental, genetic, and immune elements. Minor damage to the epithelium allows HPV infection of the basal epithelium. HPV “reprograms” cellular machinery to replicate and reproduce. The virus is then released from terminally differentiated cells that slough off from the epithelial surface (69, 70). Although most immunocompetent individuals can clear the infection within 12 months, anti-HPV antibodies are only detected 6-12 months after infection. During this delayed adaptive response, HPV can adopt intrinsic mechanisms to evade the host immune surveillance (11, 71). This can be particularly problematic for immunocompromised hosts with limited immunosurveillance.

In persistent infection, viral oncogenic expression can result in alternations to the host genome, affecting the cell cycle, and yielding a small number of aberrant cells that can avoid immune controls. Oncogenic effects on the cell cycle result in intraepithelial neoplasia, which has been extensively studied in cervical cancer (72) and is also evident in HPV-driven anogenital disease (73). Following HPV epithelial infection and integration into the host genome, oncoproteins E6 and E7 inactivate tumor protein p53 and retinoblastoma suppressor protein (pRb) (74), resulting in cellular transformation. The E6 protein is associated with p53 degradation, resulting in uncontrolled cellular proliferation and tumor growth (75). The E7 protein degrades pRb which subsequently disrupts pRb interaction with the E2F family of transcription factors. This results in increased enzyme gene replication and cell division (76, 77). Therefore, the dysfunction of tumor suppressors p53 and pRb generates hallmark features of malignancy, including uncontrolled proliferation, impaired apoptosis, and chromosomal instability (69). E7 can also act as a mutator in the mitotic process resulting in mitotic abnormalities, such as unaligned or lagging chromosomes, and breaks in chromosomal structure, resulting in further destabilization of the host cell genome (78). Furthermore, HPV prevalence is not only due to persistent infection, HPV infection of one strain increases the risk of acquiring a new HPV infection. It has been reported that 20% of men infected with one type of HPV strain, exhibited a higher number of new HPV strains on follow-up. Therefore, an individual with more than one HPV strain has an increased risk of developing HPV-associated disease (79).

### 3.3 Immune responses in HPV clearance

During HPV infection, innate immune cells act as the first line of defense, with the recruitment of pro-inflammatory and cytotoxic immune cells from the adaptive immune system used to eliminate viral particles. This results in the activation of effector cells, interferon secretion, and the release of pro-inflammatory cytokines and chemokines. Antigen-presenting cells (APCs), such as LCs and DCs, identify viral particles *via* their toll-like receptors (TLRs). HPV gene expression is confined to the keratinocytes and resolution of infection requires antigen cross-presentation by APCs and cross-priming of naïve CD8^+^ T cells for subsequent infiltration of effector T cell responses (80). This expansion of HPV-specific T cells orchestrates the adaptive immune response. Additionally, B cells are important for producing neutralizing antibodies against HPV major capsid protein L1 and aid APCs to encourage cytotoxic CD8^+^ T cell proliferation.

T cells play crucial roles in preventing chronic infection. During initial infection, CD8^+,^ T cells target viral proteins E6 and E7. In subsequent infections, the immune memory of early antigen-specific T cells eliminates virally infected cells to prevent disease establishment. This has been demonstrated in cervical cancer, where a lack of HPV-oncogenic specific CD8^+^ T cells has been associated with high-grade cervical intraepithelial neoplasia (CIN) (81). Women with active HPV16 infection demonstrated a robust cytotoxic CD8^+^ T cells response and rapid HPV clearance. Both CD4^+^ and CD8^+^ T cell responses were associated with HPV-E6-related lesion regression, while E7 regression has been attributed solely to a CD4^+^ T cell response (82–85). Therefore, E6 and E7 elicit different immune responses and recruit different T cell subsets, which could be highly dependent on HPV type. Although HPV16 and HPV18 are the predominant HPV types associated with high-grade neoplasia, E6 and E7 expression in other HR-HPV strains may elicit different immune responses, which are as equally severe in immunocompromised people and requires further investigation.

### 3.4 HPV progression to invasive cancer

A hallmark of HPV infection is its effectiveness in evading immune recognition. Viral replication is exclusively within the intraepithelial site with no induced cytolysis, viremia, cell death, or inflammation associated with replication and release (70, 86). Although effective viral clearance and HPV resolution are achieved by an effective cell-mediated immune response, in some cases, the HPV genome can lay dormant in cells evading immunosurveillance. Animal models have demonstrated that during lesion regression, when no active infection is evident, infected cells containing viral DNA persist in a latent state without viral gene expression. Although the virus is inactive, host factors including changes in hormone levels or immune suppression can cause HPV reactivation (70). HPV16 has a longer duration of persistence compared with other HPV types, which may contribute to its strong association with neoplastic development (87, 88). Failure to develop effective cell-mediated immune responses to control the infection results in high-grade intraepithelial neoplasia. Accumulation of genetic abnormalities can cause progression to invasive carcinoma. It has been shown that some cervical cancer patients lack antigen-specific T-cell responses, which may be due to an inability to recognize viral antigens. However, a clear link between HLA type and factors of host susceptibility to developing carcinoma requires further elucidation (12, 89, 90).

HPV can downregulate immune signaling pathways within keratinocytes, altering the innate and adaptive immune responses. This ceases the production of pro-inflammatory cytokines, such as type 1 interferons, that drive the inflammatory response and are important signals for the activation and migration of APCs. Transcriptomic analysis has shown oncogenic downregulation of genes with antiviral effects, such as IFIT1 and MX1, as well as those crucial for apoptosis (TRAIL and XAF1), IFN-γ signaling (signal transducer and activator of transcription 1; STAT1) and pathogen recognition receptors (TLR3, RIG-I, MDA5) (91). HPV16 E6 expression disrupts Tyrosine kinase 2 (TYK2), an intracellular enzyme important for mediating immune signaling and inflammatory pathways essential for maintaining normal immune responses. This affects the Janus kinase (JAK)/STAT signaling pathway, which has been associated with cancer progression and metastatic development and is implicated in the development of cervical cancer. The STAT pathway is associated with essential cellular mechanisms including cell proliferation, invasion, survival, inflammation, and immunity (92). Induction of STAT3 in transformed keratinocytes drives the production of the cytokine IL-6 that, in a paracrine manner, causes STAT3-associated induction of CCL2 in monocytes. This potent monocyte-attracting chemokine skews the inflammatory microenvironment, resulting in a regulatory milieu by attracting anti-inflammatory macrophages and localized regulatory T cells (Tregs) (93, 94). High CCL2 expression has been associated with poor cervical cancer outcomes, while IL-6 production downregulates CCR7 expression on activated APCs. This inhibits migration-promoting chemokines essential for homing to regional lymph nodes (95).

E7 interferes with IFN-regulatory factor 1 induction, whilst both E6 and E7 have been shown to reduce E-cadherin surface levels. This inhibits the effective migration and abundance of LCs within the active lesion vicinity (96–99). Furthermore, CCL20 is an important chemoattractant produced by keratinocytes to recruit LCs *via* the CCR6 receptor. LCs are one of the most important tissue-resident APCs, initiating T cell priming during HPV infection. E6 and E7 have been shown to suppress the NF-κB pathway, implicated in chronic inflammation in cancer, specifically affecting transcription-factor dependent CCL20 induction, promoting APC recruitment into active lesion sites (100). This modulation of signaling pathways by HPV disrupts the cell cycle, resulting in an immunosuppressive and tumor-promoting environment. The developing HPV-driven tumor cells can upregulate immune checkpoint molecules, such as PD-1/PDL1, and CTLA-4, which negatively regulate cytotoxic T cells, blocking anti-tumour specific responses. Overall, effective viral evasion strategies coupled with the disruption of crucial immunosurveillance pathways enable HPV to “hide” within host cells and skew the surrounding microenvironment towards immune tolerance.

## 4 HPV infection in primary, secondary and acquired immunodeficiencies

In immunocompromised individuals, persistent and extensive manifestations of HPV infection can result from an inadequate immune response. This includes the development of non-regressive lesions and/or progressive papillomas, leaving individuals in a pre-cancerous state and at an elevated risk of developing HPV-driven malignancies (101, 102).

### 4.1 Primary immunodeficiencies

For individuals with PIDs, HPV infection can clinically manifest as benign warts, skin squamous cell carcinomas (SCC), or HPV-driven malignancies (103). PIDs with HPV clinical implications include epidermodysplasia verruciformis (EV), WHIM (warts, hypogammaglobulinemia, infections and myelokathexis) syndrome, DOCK8 mutations, GATA binding protein 2 (GATA2) mutations, and severe combined immunodeficiency (SCID) (103, 104). In immunocompetent individuals, B-HPVs, such as HPV5, HPV8, and HPV9, infect cutaneous sites but present as asymptomatic infections (105). In the setting of specific PIDs, clinical symptoms can be characterized by body surface involvement of cutaneous warts, including common warts (verrucae vulgaris), plantar warts (verrucae plantaris), and flat warts (verrucae plana) (103). Key PIDs associated with a high risk of progressive HPV disease are characterized by defects in NK cell and CD8^+^ T cell counts as well as CD4^+^ T cell lymphopenia. Although reported in detail (103, 104), key PIDs are summarised below.

#### 4.1.1 Epidemodysplasia verruciformis

EV is characterized by mutations in the EVER1 and EVER2 genes. EVER has been implicated as a natural HPV barrier within keratinocytes and immune cells. EVER1/2 are zinc-transporting proteins expressed on immune cells including T cells, B cells, NK cells, and endothelial and myeloid cells (106). EVER1/2 encodes highly conserved membrane proteins in the zinc-transport complex, regulating zinc homeostasis and therefore limiting zinc availability to HPV-infected keratinocytes. Maintaining optimal zinc levels for cellular functions reduces viral replication and ensures an adequate anti-viral response (107). The specific roles of EVER1 and EVER2 in HPV-related disease focus on their interaction with zinc transporter 1 and its ability to drive activator protein 1 (AP-1) transcription activity. EVER dysfunction increases zinc concentration, activating AP-1, an important transcription factor for the HPV life cycle, aiding replication (107, 108). Zinc imbalance facilitates α-papillomavirus and B-papillomavirus pathogenesis, enhancing the expression of E6 and E7 (107). In EV, even LR-HPV can cause persistent disease leaving these individuals at high risk of HPV-driven SSC (109).

Tumour necrosis factor alpha (TNF-α) overproduction in EV individuals has been implicated in SCC development. TNF-α is a central cytokine important in host defense again viruses by triggering apoptosis with EVER proteins. EVER2 is a particularly important sensitizer to TNF-α-induced apoptosis. Contrary, the TNF-α signaling pathway can be harmful as it can switch from a pro-apoptotic to a pro-survival environment, thus promoting tumor progression, and metastasis (110). EVER2 is integral in preventing the pro-survival TNF-α signaling pathway by binding to TNFR-associated death domain protein (TRADD), preventing downstream pro-survival pathways and promoting TNF-α-induced apoptosis (111). Host vulnerability to persistent disease is hypothesized to be due to defects in local TNF-α signaling (111), zinc transport dysfunction (108), and increased expression of transcription factors that enable HPV replication in the absence of functional EVER protein (107).

EVER is also implicated in keratinocyte participation in local inflammatory reactions within the skin. This is through local skin secretion and responses to growth factors, various cytokines such as TNF-α, IL-6, IL-19, and chemokines, such as TFG-β and IL-8 (107). T cells, macrophages, and keratinocytes secrete pro-inflammatory IL-6, enhancing inflammation in response to pathogens (112, 113) while IL-8 aids in the migration of circulating neutrophils into tissues (114). In EVER2 -/- keratinocytes, it has been reported that production of IL-6 was reduced while IL-8 levels increased compared to wildtype. The changes in signaling between keratinocytes and immune cells could affect the ability to clear persistent lesions (115), however further investigation into these mechanisms is required.

#### 4.1.2 CXCR4 deficiency-WHIM syndrome

Warts, hypogammaglobulinemia, infections, myelokathexis (apoptosis of mature myeloid cells in the bone marrow) syndrome (WHIM) is associated with CXC chemokine receptor 4 (CXCR4) deficiency, with HPV-related disease being a major clinical feature. Manifestations include cutaneous warts typically on the hands, feet, and trunk, along with neutropenia, and in some cases lymphopenia (103). Papillomas can also develop in the genitalia and progress to neoplastic lesions and carcinoma (116, 117).

CXCR4 is a transmembrane receptor on leukocytes, endothelial cells, and stem cells, and is involved in important immune signaling pathways essential for HPV control (118). JAK2 and JAK3 interaction with CXCR4 activates the JAK/STAT pathway, which is important for immune regulation (119, 120). The interaction between CXCR4 and its ligand, stromal-derived factor-1 (SDF-1), is involved in multiple downstream signaling pathways important for chemotaxis, adhesion, and accumulation of immune cells to sites of inflammation. When CXCR4 is defective, a lack of SDF-1 signals disrupts leukocyte tracking to the affected site, allowing HPV to replicate and establish disease (121, 122). LCs and keratinocytes also express SDF-1 and CXCR4. CXCR4 gain of function mutation has been associated with an increase in cell immortalization, with increases in TNF-α expression (123, 124), driving HPV-driven carcinogenesis (103, 125).

#### 4.1.3 Autosomal recessive hyper-IgE syndrome (DOCK8 mutation)

Dedicator of cytokines 8 (DOCK8) mutations cause an autosomal recessive combined immunodeficiency resulting in a range of hyper-IgE syndromes (126). People with DOCK8 deficiency are susceptible to cutaneous viral infections, including herpes simplex virus, HPV, molluscum contagiosum virus, and varicella-zoster virus. Clinical disease presents as flat and verrucous warts, which can be extensive, disfiguring, and often treatment-resistant (127). DOCK8 has been implicated in aiding leukocyte migration, with deficiency resulting in immunoregulation failure and subsequent HPV dissemination (128). Over time, lymphopenia progresses with age, affecting CD8^+^ and CD4^+^ T cells. In mice, DOCK8 deficiency impaired the suppression of thymic Tregs (129, 130). *In vitro*, CD8^+^ T cells from patients with DOCK8 deficiency had reduced proliferation to CD3/CD28 stimulation. Although rare, IFN-γ and TNF-α production can also be impaired (126). Multivariate cox regression analysis demonstrated DOCK8 was an independent positive factor in the survival of HPV-positive (HPV+) HNSCC, and positively correlated with immune cell infiltration levels (131). The expanded skin virome has been studied in DOCK8 immunodeficiency and it was found that even in the absence of clinical HPV warts, there was a high number of abundant reads (52% mean). This contrasted with other viruses such as molluscum contagiosum virus (MCV**)** here reads were increasingly low (0.4%) when clinical manifestations were absent. This biological distinction between HPV and MCV suggests that DOCK8-deficient individuals may be more susceptive to HPV carriage, and this may explain their susceptibility to the development of squamous cell cancers (132). The differences in the subclinical presence of HPV versus MCV suggest biological distinctions between these eukaryotic viruses, with patient skin demonstrating less susceptibility to MCV than to HPV. However, further investigation to address whether there is oncogenic potential HPV detected on the skin of DOCK8 deficient individuals is required.

#### 4.1.4 Severe combined immunodeficiency (IL2RG or JAK3 deficiency)

Severe chronic HPV disease is associated with severe combined immunodeficiency (SCID) caused by IL2RG (interleukin 2 receptor subunit γ) or JAK3 deficiency. IL2RG and JAK3 are important for anti-viral immunity implicated in lymphocyte development, proliferation, and survival (133). Although hemopoietic stem-cell transplantation (HSCT) has been lifesaving for those with SCID, evidence of severe HPV disease and partial immunodeficiency are still evident up to 10 years post-HSCT. SCID solely associated with JAK3 deficiency is characterized by low NK cells, which can remain depleted following HSCT. This may have significant effects on the control of the earliest stages of HPV infection, where NK cell HPV-targeted cytotoxicity predominates (134). Keratinocytes from patients with IL2RG-deficient SCID have chemokine repertoire changes, resulting in an impaired ability to recruit immune cells (135). IL-2RG is a critical component of the IL-2 receptor and is shared among the receptors for IL-2, IL-4, IL-7, IL-9, IL-12, and IL-15. IL-2RG deficiency can impact HPV eradication and control, as it is shared among a broad array of cytokine receptors crucial for optimum innate and adaptive immunity. For example, myeloid development is highly reliant on IL-15, which in turn is dependent on IL2RG. IL-2RG deficiency impairs DCs and monocyte function, disrupting adequate signals to induce a pro-inflammatory immune response and recruit CD8^+^ and CD4^+^ T cells (136, 137). Individuals with these deficiencies are at a 50% risk of developing high-risk severe cutaneous warts post-HSCT (104, 138, 139). This suggests that HSCT does not completely revert immune defects, and a better understanding is required to tease out underlying mechanisms that allow HPV clinical disease to persist (140).

#### 4.1.5 GATA2 mutations

GATA2 is a transcription factor important for hematopoiesis and stem cell progenitor maintenance (141–143). GATA2 deficiency results in the absence of major blood cell lineages, including B cells, DCs, monocytes and NK cells (141, 144). HPV is implicated in >75% of GATA2-deficient patients with 50% being at high risk of developing persistent infection and recurrent warts (140), subsequently progressing to malignant transformation. Although HSCT can have positive effects on restoring immunity, the progression of HPV prevalence and persistence post-HSCT is not well investigated, and only reverses the effects of GATA2 deficiency in approximately half of the patients (145). The clinical features of GATA2 deficiency are highly dependent on peripheral cell numbers with severe symptoms associated with more extreme cytopenia. T cells are not as affected by GATA2 mutation compared to other subsets, however, an inversion of the normal CD4:CD8 ratio, coupled with a reduction in naïve cells, is reflective of an immune repertoire of chronic viral infection.

#### 4.1.6 Serine/threonine kinase 4 deficiency

STK4 homozygous mutations cause a deficiency associated with B cell lymphopenia and CD4 lymphopenia, particularly affecting naïve T cells, and signaling pathways important for T cell survival and death (103, 104). NK cell and CD8^+^ T cell levels remain at normal levels. STK4 deficiency results in lowering the transcription factor FOX01, which plays a significant role in T cell homeostasis, with patients showing progressive CD4^+^ T cell lymphopenia. This in turn affects downstream signaling pathways, impairing homing of naïve T cells by decreasing the IL-7 receptor, CCR7 and CD62L (103). In this condition, T cells are more prone to apoptosis and Treg development and maintenance are additionally affected (146). In immunocompetent individuals, STK4 is a tumor suppressor protein. STK4 is significantly decreased in HPV-associated cervical cancer *via* HPV E6 and E7 oncoproteins suppressing STK4 mRNA and increasing YAP-dependent gene expression, which aids HPV replication and tumor progression. Therefore, defects in STK4 result in individuals being at an elevated risk of HPV-driven carcinogenesis (147).

### 4.2 Secondary and acquired immunodeficiencies

#### 4.2.1 Transplant recipients

Immunosuppressive therapy improves long-term graft and patient survival in transplant recipients. However, immunosuppressive drug regimens increase the cumulative occurrence of persistent HPV infection, including the development of pre-cancerous lesions and treatment-refractory cutaneous and anogenital warts. The development of HPV-associated warts in transplant patients has been linked to the duration of immunosuppression, with 50-90% of patients four to five years post-transplant developing warts (148). In HSCT, the development of HPV-driven cervical, vaginal, and vulvar warts leaves women at an increased risk of developing related cancer (149). A systematic review of anogenital HPV-associated cancer in solid organ transplant recipients found the highest increased standardized risk in cancers of the vulva and vagina (22.8), penis (15.7) and anus (4.9). Transplant patients with underlying HPV-associated infection and precancerous lesions were at the highest risk of developing cancer post-transplant (150).

The predominant body of evidence regarding persistent HPV infection transitioning to anogenital neoplasia and cancer is in renal transplant patients. Renal transplant studies have demonstrated that latent HPV reactivation occurring post-transplantation increased HR-HPV active infection by 27% (151–153). Additionally, prolonged CD4^+^ T cell lymphopenia has been reported in renal transplant patients that can persist for up to 10 years post-transplant, leaving individuals at an elevated risk of HPV disease persistence and allograft rejection (154, 155). Renal transplant recipients have a 65-100-fold increase in developing SCC compared to immunocompetent individuals (156, 157). In women who have undergone a renal transplant, a 5-fold increased risk of developing genital warts was observed compared to the control population (158). Within the first year of a renal transplant, the frequency of HR-HPV infection increased from 24% to 36% over a period of 6 months. The high HR-HPV viral load was positively associated with the development of HPV CIN, a cancer precursor (159). A case-control study reported that 20% of renal transplant patients were diagnosed with anal intraepithelial neoplasia (AIN) compared to 1% of controls (1). RT-PCR confirmed HPV16 prevalence in 47% of the transplant group versus 12% in the control group. Despite no evidence of anogenital disease in patients before transplant, AINs developed post-transplant in the setting of immunosuppressive therapy (160). AIN prevalence in ASCC is 20%, with established HPV infection being prevalent in 47% of transplant patients (160). Natural history studies of AIN in renal transplant recipients are predominately reported in HIV+ men who have sex with men (MSM), where more than 50% of lesions progressed from low to high-grade AIN over a two-to-four-year period (161). This response may be particular to renal transplant, with varied responses reported following other forms of transplant (162). Similarly, in a study of 1023 women following renal transplant in the Netherlands, there was a five-fold increased risk for cervical cancer, followed by a 41-fold increased risk in the vulvar and a 122-fold increased risk of anal cancer in the transplanted compared to the general population. In 91.7% of lesions, HR-HPV was detected with 54.5% being attributed to HPV16 (153).

For transplant recipients, viral oncogenicity is highly dependent on the level of required immunosuppression. The nature of immunosuppressive drugs is to block cytotoxic T cells to prevent organ rejection, however, this decrease in immunosurveillance can cause HPV reactivation and replication. *In vitro* and *in vivo* studies have shown that corticosteroids, calcineurin inhibitors (e.g. cyclosporine A) and antimetabolites (e.g. azathioprine) have a direct pro-oncogenic effect (163–165). Calcineurin inhibitors have been reported to be more potent immunosuppressors compared to other drugs such as antimetabolites, being associated with a higher risk of developing HPV disease. One study reported that ~36% of kidney transplant patients treated with cyclosporine, azathioprine, and prednisone, developed papillomatous lesions with 25% of these lesions attributed to HPV16 infection. Oral cavity lesions occurred in 14% of recipients treated with cyclosporine, which was not evident in those on an immunosuppressive regime without cyclosporine (166). In contrast, another study found a non-significant correlation between HPV status and cyclosporine use (167). There is minimal understanding of whether specific immunosuppressive drugs taken alone or in combination increase the likelihood of HR-HPV reactivation in transplant recipients. Improved knowledge around combination immunosuppressive drug regimens and their link to HPV reactivation and related diseases may lead to improved treatment protocols that reduce high-risk groups developing HPV disease.

#### 4.2.2 HPV-driven malignancy in the setting of HIV co-infection

PLWH have an acquired immunodeficiency that places them at high risk of developing HPV-associated malignancies. Antiretroviral therapy (ART) reduces the incidence of many AIDS-defining cancers, including Kaposi’s sarcoma caused by herpesvirus and EBV-driven non-Hodgkin’s lymphoma. However, any beneficial effects in reducing the incidence of HPV-driven HSILs, persistent disease, and cancer risk are not well understood. PLWH are at a higher risk of developing many types of cancers compared to the general population (14, 18, 60, 168–171). While HIV+ women have significantly higher rates of cervical CIN when compared to uninfected women (172–174), HIV+ MSM have an increased risk of developing HPV-associated AINs (170, 175, 176). However, there is a limited understanding of HPV type and distribution. In Kenya and South Africa, HPV16/18 prevalence was not significantly different between HIV+ and HIV-negative (HIV-) women with cervical carcinoma (177). However, higher CD4^+^ T cell counts were negatively correlated to infection with multiple HPV types (177). CD4^+^ T cell counts of <200 cells/μL are strongly associated with HR-HPV infections as well as genital wart development (178–181). Therefore, even though the HPV distribution may not differ in the presence of HIV, CD4^+^ T cell lymphopenia may facilitate HR-HPV infection due to a lack of effective signaling to enable a robust cytotoxic response. While the oncogenic potential of HR-HPV is well-established, the impact of HIV co-infection on HPV and the downstream susceptibility to malignant transformation is not well understood (150).

The common underlying mechanisms of host susceptibility to HIV and HPV co-infection are centered around the disruption of epithelial integrity. It has been postulated that HIV and HPV co-infection occurs locoregionally within an individual, but not within the same cells. HIV infects CD4^+^ immune cells that can elicit disruptions to epithelial integrity, increasing the ability of HPV to reactivate or infect the host (182). Mucosal disruption is required for HIV virions to breach the mucosal barrier in the anogenital tract to target cells for viral replication, which can subsequently provide a pathway for HPV co-infection and progression to disease establishment and malignancy. It is hypothesized that mucosal epithelial cells upregulate inflammatory cytokines, such as TNF-α and TGF-β (183), and downregulate E-cadherin and tight junction proteins, allowing permeability and access for HIV and HPV to invade (184–186). Depending on the period between HIV establishment and HPV infection, the immune system, specifically CD4^+^ T cells, may already be affected and present at low numbers, increasing host susceptibility to persistent HPV infection.

##### 4.2.2.1 HIV infection can increase host susceptibility to HPV invasion

While damage to epithelial integrity can result in susceptibility to HIV and HPV co-infection, active HIV infection may also increase susceptibility to HPV. A reduction in key innate molecules, including B-defensin-2 and thrombospondin, has been proposed due to the interaction between the two viruses,resulting in an increased risk of HPV-induced malignancies (187, 188). Defensins are important for viral inactivation, and the recruitment of T cells and neutrophils (189, 190). In CIN and invasive cervical SCC, B-defensin-2 is lower compared to the normal ectocervical epithelium (189). A similar pattern in thrombospondin levels is seen in invasive SCC (188). The HIV protein Tat is also implicated in increasing the gene expression of both HIV and other DNA viruses (191, 192). Disruption of tight junctions by HIV Tat and glycoprotein 120 (gp120), enables HPV to penetrate the basal layer of the oral epithelium (193). HIV Tat may increase the expression of E6 and E7 proteins, as well as E2, which is important for HPV replication (194) and has been described in HPV-associated cervical and oral SCC (195–197). Additionally, Tat can upregulate HPV-associated oncogenesis and reduce p53 protein levels, as well as re-activate dormant HPV (195, 198). Therefore, HIV results in pathogenesis that increases the likelihood of HPV disease in parallel.

Although many HIV-associated comorbidities have declined with ART, HPV disease burden remains high, specifically for anal and cervical SSC (199, 200). There is conflicting data on the impact of ART on HPV infection. Women living with advanced stages of HIV undergoing ART had a significant reduction in oncogenic HPV infection, prevalence, and incidence, whereas another study contradicted this outcome and suggested that ART was associated with increased lesions, warts, and oral HPV persistence (201, 202). Swiss patients (6%) undergoing highly active ART for four years, continued to have low CD4^+^ T cell counts (<200 cells/μL), with 50% unable to reach the clinically acceptable 500 cell/uL count (203). Prolonged ART in HIV-infected individuals may not improve HPV-specific immunity, resulting in HPV persistence and the development of neoplasia (204, 205). Additionally, although T cell reconstitution occurs, it may be functionally dysregulated. Such T cell dysregulation includes Treg dysfunction, poor antigen presentation by dendritic cells, as well as a skewing toward an inflammatory T helper 1 response (206).

##### 4.2.2.2 Active HPV infection may increase the likelihood of HIV transmission in the host

While there is growing evidence that HIV infection can cause immune dysfunction resulting in opportunistic HPV disease, it has also been suggested that HPV can in turn increase the risk of HIV transmission. A meta-analysis of the effect of HPV infection on HIV susceptibility demonstrated that HPV infection is associated with a two-fold increased risk of HIV acquisition (207, 208). In sub-Saharan Africa, women with prior HPV cervical infection were at 2.4 times higher risk to acquire HIV than the control cohort (209), whilst a separate study found that HPV+MSM had a 3.5-fold increased risk of HIV seroconversion (210, 211). MSM with a CD4^+^ T cell count <200/μL had a higher risk of developing HSILs, with HIV+MSM having an increased risk of developing HPV-associated anal SCC.

Increased HIV susceptibility to an HPV-infected host has been linked to high HPV viral load, inflammatory cytokines at mucosal surfaces, mucosal barrier damage, CD4^+^ T cells in anogenital mucosa, and expression of neutrophil proteases. During HPV infection, lesion regression is marked by a high CD4^+^ T cell count within the stroma and epithelium, which potentially provides HIV with an opportunity to infect their target cell (212). Inflammatory mediators, such as MCP-1, IL-8 and LP-1, implicated in NK and T cells responses to wart regression, are important for HPV clearance but also associated with high HIV acquisition (213–215). Recruitment of neutrophil proteases and pro-inflammatory cytokines disrupts normal epithelial cell differentiation and epithelial barrier and integrity. Although HIV is known to primarily enter cells *via* CD4, HIV utilizes other common receptors, including CXCR4 (216) and CCR5 (212, 217). The HIV envelope glycoprotein binds to these receptors to facilitate HIV entry into cells, followed by membrane fusion and viral internalization where replication occurs (191). In the presence of HPV, a relative abundance of CD4^+^ T cells, DCs and macrophages that express CXCR4 and CCR5 provide HIV entry. DCs are known to capture HIV antigens and migrate to the lymph node for antigen presentation, however prolonged contact with autologous CD4^+^ T cells, can facilitate HIV infection (218). Therefore, these potential mechanisms and favorable environments during HPV infection can pave the way for successful HIV transmission.

## 5 HPV-driven cancers

### 5.1 HPV detection methods

Various detection methods are used to identify HPV to account for differential biomarkers and histopathological differences [**Table 1**; adapted from (219)]. Molecular detection methods and screening tools for specific HPV-driven cancer types have been previously summarised (220). In HPV-associated OPSCC (HPV+OPSCC), high levels of p16 and wild-type p53 are indicative of HPV infection. Immunostaining of p16 (associated with HPV-16) can be used as a marker to identify HPV-implicated cancers (221). This is of particular importance in OPSCC which can be caused by HPV or occur independently of HPV. HPV-negative OPSCC (HPV-OPSCC) can be identified through mutation in p53 and poor p16 expression due to deletion, mutation, or hypermethylation (222). In HPV+OPSCC, p16 overexpression can also be a result of other non-HPV-related pathways. Consequently, p16 staining is commonly used in conjunction with other methodologies to identify HPV+OPSCC accurately (223).

**Table 1:** HPV methods of detection used to identify HPV genotype.

Methods	Specificity	Sensitivity	Advantages and disadvantages
**Southern Blotting Assay**	High	High	Ability to differentiate between episomal and integrated DNA. *Not easily applied to FFPE samples*
**ISH**	High	Low	Ability to differentiate between episomal and integrated DNA. *Low sensitivity*
**HPV PCR**	High	Low	Cost-effective *Does not quantitate viral load or inform whether the virus is active (low sensitivity).*
**Real-time PCR**	High	High	Ability to differentiate between episomal and integrated DNA. *Not easily applied to FFPE samples and is labor-intensive.*
**Reverse Transcriptase PCR**	High	High	High sensitivity *Time-consuming*
**p16 Immunostaining**	Low	High	Identifies marker of transcriptionally active virus. *Low specificity*
**Signal Amplification Methods**	High	Low	Methodology easily performed (user-friendly) *May produce false positives (low sensitivity)*

### 5.2 Predominant HPV-driven cancers in immunocompromised individuals

#### 5.2.1 Cervical cancer

Cervical cancer is the fourth leading cause of cancer in women globally, accounting for 342 000 deaths worldwide in 2020 (224). The initial stages of cervical cancer can be asymptomatic prior to progression, where symptoms then include vaginal bleeding, pelvic pain, and dyspareunia (26). Immunocompromised individuals are at the greatest risk of developing non-regressive HPV-related lesions which can transition to cancer. Compared to uninfected women, women living with HIV have a two-fold increase in developing cervical cancer (26, 225, 226). In immunocompetent individuals, cervical cancer can take 15-20 years to develop, compared to 5-10 years in the setting of HIV co-infection. Cryotherapy or thermal ablation can be used to treat pre-cancerous lesions. HPV testing of women aged 30-65 years, with cervical screening occurring from the age of 21 or following the onset of sexual activity can aid in the early detection of HR-HPV genotypes and result in early intervention. If diagnosed with HIV, cervical cytology is recommended every six months from the year of diagnosis, with annual screening thereafter following no evidence of HPV infection. In solid organ transplant patients, cervical cancer screening is also performed annually (227). A colposcopy, with or without a lesion biopsy, is conducted if abnormal cervical cells are evident. A cervical cancer diagnosis is made through histopathological examination with tumor size and disease metastasis defining the cancer stage (73).

#### 5.2.2 Anal squamous cell carcinoma

HPV is detected in 80-90% of anal cancers, with HPV16 being the predominant type (228). The prevalence is highest in Australia, North America and regions of Europe (229). Like cervical cancer, anal carcinoma develops from lesions of varying cytological and histological severity, known as AIN. While low-grade AIN regressed in most immunocompetent individuals, high-grade AIN can progress to invasive cancer (230, 231). Persistent HR-HPV infection causes pre-cancerous lesions in immunocompromised individuals that do not regress due to inadequate adaptive immunity. These dysplastic lesions can progress to ASCC (232).

Despite the widespread availability of ART, the incidence of ASCC is continuing to increase (233). MSM and PLWH exhibit a 20-fold and 30-fold increased risk of ASCC, respectively (171), and HIV+ MSM have a 50-150-fold increased risk (232, 234, 235). In the natural history Study of the Prevention of Anal Cancer (SPANC), HIV+ and HIV- MSM (≥35 years) were enrolled to explore anal HPV infection epidemiology and cytological and historical abnormalities. HSIL incidence was strongly associated with younger age and persistent HR-HPV infection. Conversely, HSIL clearance was associated with smaller lesions, AIN 2, and a lack of HR-HPV infection (171). HPV16 and HPV18 showed the highest prevalence in MSM, however, HPV31, HPV22, and HPV58 also demonstrated increased incidence (>2.0/100 person-years). Women with a history of cervical dysplasia and transplant patients are also at an elevated risk. Renal transplant recipients have been shown to have a 10 times greater relative risk of developing ASCC than the general population (236).

ASCC screening involves high-risk populations undergoing anorectal exams where anal swabs can be collected for HPV genotyping and the identification of atypical cells. Screening aids in the early diagnosis and treatment of precancerous lesions prior to cancerous progression. Identification and characterization of early-stage cancer are facilitated by anal cytology and high-resolution anoscopy (HRA), which obtain directed tissue biopsies (176, 237). HRA is limited in its application due to restricted views in cases of uneven lesion topography, necessitating parallel use of digital anorectal exams. ASCC screening is not as established as cervical cancer screening, with little investigation into the effectiveness of routine surveillance and the treatment of high-grade AINs (60). With the rising prevalence of ASCC in immunocompromised individuals, additional research is required to develop effective screening strategies to accurately identify high-risk lesions in earlier stages where intervention mayimprove prognosis.

#### 5.2.3 Oropharyngeal squamous cell carcinoma

Oropharyngeal SCC is associated with significant morbidity and mortality. The global, incidence of OPSCC in 2020 was 3,777,713 with the highest prevalence in Asia, Europe, and North America (26). OPSCC affects the upper aerodigestive tract, including the tonsils, the base of the tongue, and the soft palate. Unlike most HNSSCs, which are associated with smoking and alcohol consumption, a biologically distinct subset of OPSCC arises from HPV. HPV+OPSCC represents 15-20% of HNSSC associated with HPV, with 95% of HPV+OPSCCs attributed to HPV16 (238). Over the past two decades, there has been an increase in HPV+OPSCCs, and a decrease in HPV-OPSCC prevalence due to lifestyle changes including a decrease in smoking rates (239–241). Compared to HPV-OPSCC, HPV+OPSCC occurs in younger individuals, with less exposure to alcohol and smoking, and a greater number of sexual partners. Immunodeficient individuals can also be at elevated risk, with PLWH demonstrating a two to six times increased risk of developing HPV-associated HNSCC (242). Immunosuppression is another major risk factor for SCC development, with organ transplant recipients having a 65 to 100-fold increase in risk compared to the general population. Currently, there is no standard screening regime or treatment for HPV+OPSCC or oral cancer, with therapy based on tissue site rather than cancer pathophysiology. Like anal cancer, optimal screening and therapeutic regimens may depend on the underlying pathophysiological mechanisms. CD8^+^ tissue-resident T (CD8^+^ T_RM_) cell populations in OPSCC are correlated with better patient survival and the underlying cellular and molecular mechanisms require further investigation (243). Further understanding of differences between HPV+OPSCC and HPV-OPSCC has the potential to inform novel host-directed therapies in this area.

#### 5.2.4 Vulva cancer

VC accounts for 5% of gynecological cancers with HPV16 and HPV18 implicated in non-keratinizing vulva SCC (26). HPV-positive VC is diagnosed in young women, with immunosuppression being a significant risk factor (244) transplant patients have a 100-fold increase in VC incidence. Immunosuppressed patients develop VC precursor intraepithelial neoplasia, which displays hallmarks of severity i.e. multifocal lesions with extensive disease (245). In women living with HIV, vulvar intraepithelial neoplasia (VIN) development was 29 times more likely compared to the general population, with a 3.3-fold increase in the risk of persistent VIN post-treatment (246–248).

### 5.3 Clinical management of HPV-driven cancer

Clinical management of HPV-associated malignancies relates to the site of neoplasm. HPV-associated cancer treatment strategies are multimodal and can include monotherapies or combinations of traditional surgery, chemotherapy, radiotherapy, and targeted immune therapy (249, 250). Many patients with early nodal involvement require initial surgical removal and radiation therapy (251), whereas, in more advanced tumors, treatment is centered around radiochemotherapy (252). Therapeutic regimens for several malignancies are currently approached in the same manner irrespective of HPV status, and it is unclear if there is treatment equivalence in people who are co-infected with HPV and HIV (253). PLWH have shown improved outcomes from immune checkpoint inhibitor blockade (i.e. PD-1 inhibition) however, the detailed cellular and molecular mechanisms underlying this response are unclear.

The most comprehensive data for PD-1 inhibitors derive from the use of pembrolizumab (254) and nivolumab in HNSCC (255). In a KEYNOTE trial in active metastatic HNSCC, cetuximab (a drug that restricts tumor growth) with chemotherapy was compared with pembrolizumab monotherapy or pembrolizumab in combination with chemotherapy. In both pembrolizumab treatment groups, HNSCC exhibited improved overall survival when compared to cetuximab with chemotherapy. Patients on pembrolizumab monotherapy had a median overall survival of 14.9 months, compared to 10.7 months in the cetuximab with chemotherapy group. Similarly, patients on pembrolizumab with chemotherapy showed a median overall survival of 13 months versus 10.7 months in the cetuximab with the chemotherapy group. Despite these data suggesting the benefit of using pembrolizumab, HPV status was not reported among patients (256). In the KEYNOTE01 trial, using pembrolizumab in active HNSCC, HPV+HNSCC patients showed better progression-free survival (4 months) versus HPV-HNSCC (2 months) (255). A comprehensive summary of immunotherapies in HNSCC patients and differences in immune responses has been previously reviewed (249).

Similar clinical trials are ongoing in cervical cancer and ASCC. In cervical cancer, pembrolizumab was approved in 2018 based on a phase II study (KEYNOTE 158 -NCT02628067). This involved 98 patients with recurrent and/or metastatic cervical carcinomas (257), however, results did not show the same level of improvement as seen with HNSCC. Reports of the effects of pembrolizumab in cervical cancer are sparse and a better understanding is required amidst ongoing trials (KEYNOTE-826, phase III trial, NCT03635567) (258). Several clinical trials including NCT03233711, NCT02314169, and NCT02919969 have investigated PD-1 blockade in ASCC, and have demonstrated anti-tumor efficacy (259, 260). However, much of this work has not explored the efficacy of PD-1 blockade in the context of HPV and HIV coinfection. Further understanding of the molecular pathways underlying the responses to immune checkpoint blockade, resulting in tumor reduction is required (261).

The routine screening of pre-cancerous lesions remains the most effective evaluator of cancer development risk. Following cancer development, clinical management of HPV-related malignancies in immunocompromised people is multifactorial and dependent on cancer type and stage. In the ART era, alternative therapies, such as chemotherapy, have been increasingly offered alongside targeted therapy. A significant challenge is designing a treatment plan that will minimize the risk of leukopenia and immune dysfunction, increasing the risk of other opportunistic infections. For PLWH, ART is continued through cancer management to preserve adequate adaptive immunity. Surgery is useful if lesions are located in specific areas with well-defined margins and can involve electrodesiccation to destroy tumor cells. Chemotherapeutic drugs and HIV medication are metabolized through cytochrome p450 within the liver, with some chemotherapy agents regulating or inhibiting this enzyme which can in turn decrease treatment efficacy or increase toxicity. As with chemotherapy, ART can also interfere with the pharmacology of targeted therapy (262, 263).

## 6 Advances in HPV therapeutic strategies

### 6.1 Current conventional therapies and treatment plans

The 9-valent HPV (9vHPV) prophylactic vaccine targets HPV types 6, 11, 16, 18, 31, 33, 45, 52, and 58 (264) and has demonstrated efficacy in reducing HPV incidence in immunocompromised people (170). he 9vHPV vaccine covers the major HR-HPV and LR-HPV genotypes responsible for 70% of cervical cancers and 90% of other HPV-associated cancers (265, 266). Immunogenicity data suggest that a two-dose regimen with a 6-month interval is effective in immunocompetent individuals 16 years old and under, while a three-dose regime is recommended for those older than 16 years old (170, 267). Although a gender-neutral HPV vaccination regime inclusive of both boys and girls is beginning to be implemented in some upper-income countries (268), the major challenge lies in lower-income countries where vaccinating young girls is still a challenge due to limited resources. Furthermore, despite this new gender-neutral regime taking place, there is a significantly lower number of boys getting vaccinated, with 44% and 5% of boys being vaccinated in high-income and low/middle-income countries respectively. Nevertheless, prophylactic vaccines are not effective against established HPV infection and there is a need for a therapeutic HPV vaccine or specifically targeted immunotherapy for individuals that have already acquired HR-HPV.

For immunocompromised individuals, the long-term vaccine efficacy of prophylactic vaccines is poorly understood. Currently, no approved therapeutics targeting HPV are available for use post-infection. As the incidence of HPV rises in low-socioeconomic nations, there is a requirement for any novel HPV therapeutic (prophylactic or curative) to be cost-effective, globally distributable, and equally accessible whilst covering a large proportion of HR-HPV genotypes (269). As HPV poses a significant lifetime risk of associated cancer development within an immunocompromised host, a therapeutic capable of directly targeting HPV within infected cells or boosting the host’s immune response to HPV is essential. Whilst current vaccination strategies utilize the L1/L2 capsid proteins as targets, this approach fails to account for limited L1/L2 expression in basal epithelial tissue where carcinogenesis occurs, preventing the initiation of a strong immune response (270). While prophylactic vaccination aims to prevent infection, therapeutic vaccines aim to generate robust cytotoxic lymphocyte responses against E6 and E7 viral proteins (269). Several different therapeutic vaccines are currently in development, including protein and peptide-based vaccines, live vector-based, DNA, and specific-cell-based vaccines, which have been discussed in detail elsewhere (271).

In contrast to prophylactics, host-directed therapies for HPV-driven cancer focus on the generation of host immune responses against antigens associated with cellular transformation. Such treatments include adoptive cell transfer, genetically engineered T cell therapy [T-cell receptors (TCRs) and chimeric antigen receptors (CARs)] (272, 273), and the use of immune checkpoint inhibitors (255, 258–260). Adoptive cell transfer, involves autologous antigen-specific CTL expansion *ex vivo*, with re-delivery to the patient’s circulation (274). Although promising, the reintroduction of CTL to the host is highly dependent on immune and tumor cell signaling to maintain an effective immune response. This can result in varying levels of effectiveness. A major challenge of emerging immune or antiviral-based therapies targeting HPV is that a clear advantage over current treatment must be demonstrated whilst ensuring patient safety. Developing such therapeutics for use in immunocompromised patients has additional complexity, due to a suppressed immune response. Equal consideration for potential drug interactions with concurrent medications in both HIV individuals on ART and transplant patients on anti-rejection regimens is required. Therefore, a novel approach to the development of therapeutics is targeting HPV-specific proteins or genes, to prevent tumor establishment and cancer progression in hosts where the immune system is impaired.

A comprehensive list of therapeutic and immunotherapy-based developments has been previously summarised (269, 275), however minimal data is available for those that are immunocompromised. Despite major developments in therapeutic vaccinations and immunotherapies, a need to develop treatments that can cater to individuals that do not have an intact immune system persists. A thorough understanding of HPV-cellular interaction, virus-host interplay, and the tumor microenvironment’s role in the development of immunotherapies that are specifically effective toward HPV-transformed cells is required (66). It is likely that for immunocompromised hosts, successful and effective treatment plans will be multi-pronged, focusing on aspects such as tumor microenvironment modulation, and specific alteration of HPV-infected cells to make them less resistant to therapeutic intervention.

### 6.2 RNA-based therapeutic strategies against HPV

Whilst therapeutic regimens exist in relation to HPV, all current therapeutics premise on a functional immune system. This excludes a small yet significant range of patients from obtaining adequate care. With the exception of mRNA-based vaccination (which necessitates an immune system by definition), RNA-based therapeutics hold the unique ability to operate in severely immunocompromised patient populations.

#### 6.2.1 mRNA prophylactic vaccination

Next-generation mRNA-based vaccinations are a novel technology applicable to HPV that has shown significant promise in the fight against SARS-CoV-2 over the past two years and demonstrated safe and highly efficacious responses in typical and atypical patient populations (276, 277). Compared to traditional HPV vaccine candidates, mRNA vaccinations designed against HPV proteins lack any potential risk of causing disease, enable the regulation of immunogenicity, and evade anti-vector immunity (278). mRNA vaccination results in type I interferon (IFN-I) pathway activation and the production of pro-inflammatory cellular cytokines and chemokines, promoting a stronger immune response *via* increased APC stimulation (279). Cost remains the most significant hurdle to the widespread adoption of mRNA vaccination, as scalability and availability of suitable manufacturing facilities increase the cost per unit (280). Despite the challenges associated with initial cost, the flexibility offered by mRNA vaccination in response to novel pathogens makes them an appealing and viable avenue for therapeutic development. Investigation into the development of a single-dose prophylactic mRNA-based vaccination against HPV may also enable the greater prevention of new HPV incidences in developing regions by eliminating the need for two vaccine doses, as currently recommended by global governing bodies, reducing the load on local health infrastructure whilst improving intergovernmental responses to novel viral variants.

#### 6.2.2 Zinc finger nucleases

Zinc finger nucleases (ZFN) are engineered, modifiable nucleases consisting of a Fokl DNA-cleaving domain bound to zinc finger proteins. Two complimentary ZFN join to form an active ZFN complex capable of recognizing sequence motifs ≥24 base pairs (281). ZFN is programable by altering the amino acid sequence of each zinc finger, enabling site-directed double-stranded cleavage to suppress transcription (281).

ZFN has been demonstrated to be efficacious in the disruption of the HPV E7 protein expression *in vitro* and *in vivo*, recognizing HPV-specific genomic target sites and initiating double-stranded cleavage (282). It has been found that by targeting the E7 DNA region of HPV16+ (SiHa/CaSki) and HPV18+ (HeLa) cell lines, HPV-specific ZNF can effectively induce disruption of E7 oncogenes, leading to type-specific growth inhibition and apoptosis of HPV+ cells (282). ZFN have similarly shown promise in regulating HIV resistance *in vitro* and *in vivo* (283).

While ZFN provides numerous advantages including site-directed epigenetic modification, significant disadvantages arise. ZFN are difficult to construct, limited in target selection, and remain expensive in comparison to second-generation and third-generation genomic editing technologies, restricting their widespread adoption for use in HPV treatments in developing regions (284, 285).

#### 6.2.3 Clustered regularly interspaced short palindromic repeat

Since its first reports in 1993, CRISPR systems have quickly developed to become one of the most powerful tools associated with the field of genomics (286). CRISPR-associated (Cas) proteins enable the precise epigenetic regulation of DNA through either insertion, deletion, or mutation of the genomic target. Many Cas proteins are nucleases that cleave the double-stranded DNA at the site of the genomic target, however, it is possible to affix various classes of effectors proteins (287). CRISPR/Cas9 specifically locates and binds a protospacer adjacent motif (PAM) within the host genome (288). An associated guide RNA interrogates the DNA strand to locate the complementary genomic target. Following the location of the specified target, the CRISPR/Cas system induces epigenetic modification to alter gene transcription and associated protein expression (289). Nuclease sites of the Cas protein cleave the DNA, which can be repaired *via* end joining, resulting in mutagenesis, or homology-directed repair (290).

CRISPR/Cas9 technology has shown significant promise in the suppression of virally expressed HPV E6/E7. CRISPR/Cas9 mediated frameshift knockout of the E7 oncogene has been shown to significantly inhibit aberrant cell proliferation of HPV18+ Hela and HPV16+ SiHa-associated cancerous activity *in vitro. In vivo* confirmation using micelle delivery, CRISPR/Cas9 within xenografted mice demonstrated significant E7 knockout (291, 292). Additionally, CRISPR/Cas9 systems developed against E6 and E7 have been shown to restore the protein expression of p53 and phospho-pRB (292). Increased cellular levels p56 and phospho-pRB mitigate the hallmarks of HPV cancerous lesion progression by: (I) reducing cellular proliferation (II) ceasing DNA synthesis (III) halting cell cycle progression (IV) inducing apoptosis (V) down-regulating expression of E2F-1 and bcl-2 (293). These findings suggest that CRISPR/Cas9 systems hold the potential to strongly inhibit tumorigenesis post HPV genome integration and serve as a viable therapeutic avenue, although further investigation into unintended off-target effects is required for advancement to clinical trials. Further, the delivery of CRISPR/Cas9 therapeutics requires not just a gRNA molecule, but also the large Cas9 protein or mRNA, increasing the manufacturing complexity, cost and size of the delivery carrier.

#### 6.2.4 Ribonucleic acid interference

**Figure 3:**
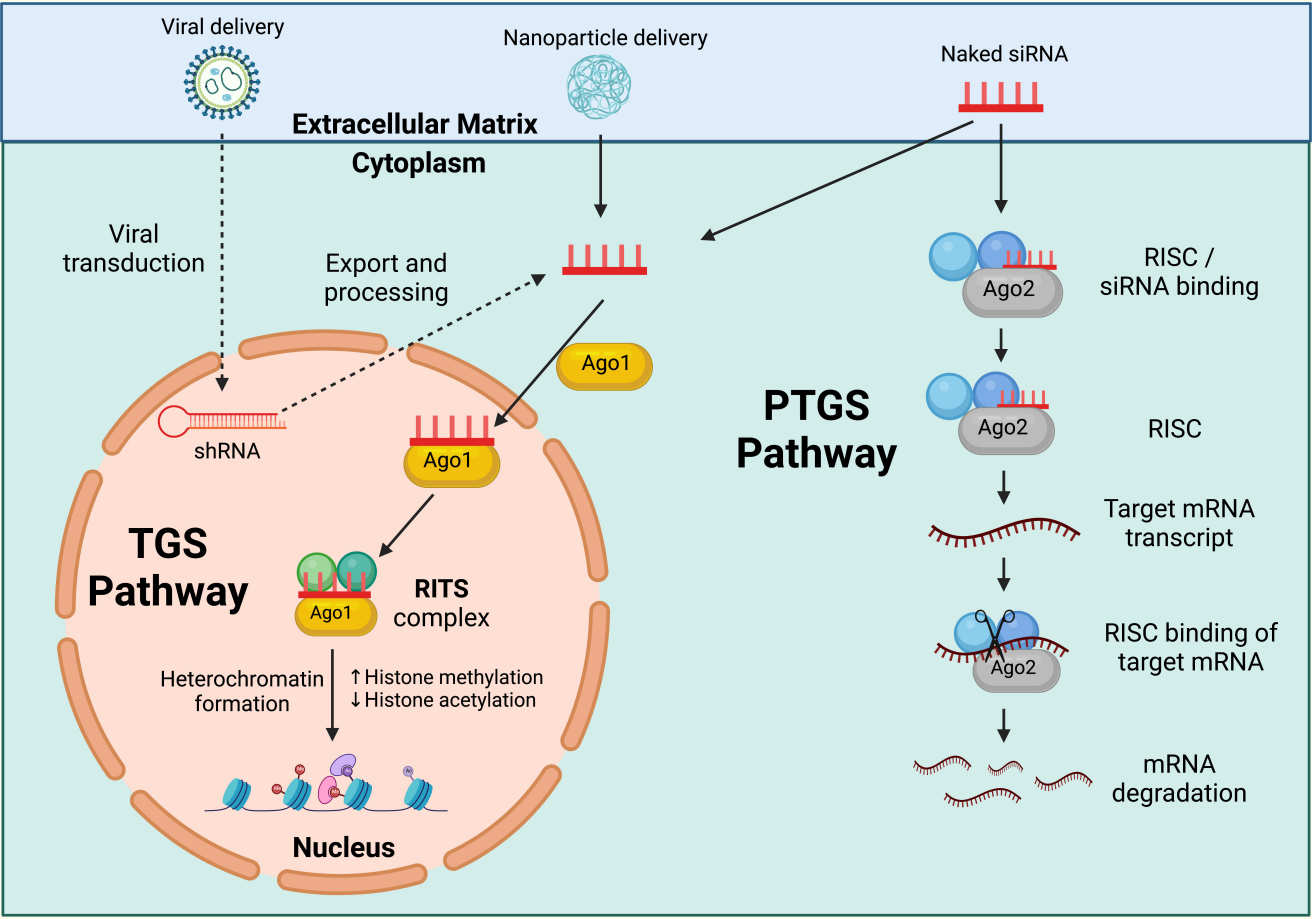
RNAi pathways. Post Transcriptional Gene Silencing (PTGS) inducing short interfering ribonucleic acid (siRNA) operate via the actions of the ribonucleic acid (RNA) induced silencing complex (RISC), a multiprotein nuclease complex comprised of Argonaute-2 (Ago-2), Dicer, TAR RNA binding protein, protein kinase interferon-inducible double stranded RNA dependent activator, and GW domain protein 182 in humans. The antisense strand binds Ago-2 and is incorporated into the RISC complex. The antisense strand guides the complex to the targeted mRNA product, where Ago-2 facilitates the cleavage of, and subsequent degradation of the messenger ribonucleic acid (mRNA). Transcriptional Gene Silencing (TGS) inducing siRNA operate via the actions of the RNA induced transcriptional silencing (RITS) complex, a multiprotein complex comprised of Argonaute-1 (Ago-1) and other, yet unidentified, proteins in humans. The antisense strand binds Ago-1 and is incorporated into the RITS complex, where it guides the complex into the cell nucleus and targets the gene promoter. Heritable epigenetic modifications result in compaction of chromatin structures surrounding the target site, preventing the transcription of gene products, and silencing expression. Created with BioRender.com.

Ribonucleic acid interference (RNAi) is a promising gene-silencing technology that can be applied to HPV therapeutic research to restrict viral replication and improve host clearance. RNAi is a highly conserved, biological process that can operate *via* two distinct pathways in response to double-stranded ribonucleic acid (dsRNA) (1): *via* the targeting of transcribed messenger ribonucleic acid (mRNA) products within the cell cytoplasm, termed post-transcriptional gene silencing (PTGS); or 2) by directly targeting the gene promoter region located within the cell nucleus, in a process termed transcriptional gene silencing (TGS) (294). These mechanisms can be used to downregulate or impede gene expression for therapeutic application (**Figure 3**). Whilst numerous dsRNA duplex classes can operate *via* RNAi, short interfering ribonucleic acid (siRNA) is one class that can act *via* both the PTGS and TGS RNAi pathways (295, 296). siRNAs are 19-24 base pair (bp) double-stranded (ds) oligonucleotides, comprising of a 3’-5’ active guide (antisense) strand and 5’-3’ passive passenger (sense) strand (297).

PTGS-mediated siRNA targeting HPV oncogenic proteins E6/E7 significantly decreases the levels of mRNA transcripts associated with infection. Downregulation of viral gene expression has been demonstrated *in vitro* within the HPV16+ SiHa and CaSki, and HPV18+ Hela cell lines (296, 298, 299). Reduction in viral gene expression enables increased intercellular levels of p53 and pRb, which serve to mediate the effects of HPV. Findings obtained in commercial cell lines were replicated *in vivo* using nude mice, where treatment with PTGS-mediated siRNA targeting mRNA associated with E6 or E7 significantly reduced tumor size, weight and oncogenic expression (300–303). PTGS siRNA is highly effective, but restrictive as only a singular gene product can be targeted per engineered strand, compared to TGS-mediated promoter-targeted siRNA, which can target multiple gene products under the regulation of the same promoter.

TGS-mediated siRNA targeting viral gene promoters have similarly shown strong efficacy in reducing E6/E7 mRNA expression using a single siRNA targeting the site of transcription initiation (304). TGS siRNA targeting the common HPV E6/E7 promoters p97 (HPV16) or p105 (HPV18) have shown significant down-regulation of mRNA associated with E6 and E7 *in vivo* (305–307). Like PTGS siRNA, the reduction in viral gene expression enables increased intercellular levels of p53 and pRB, which serve to mediate the carcinogenic effects of HPV infection. A striking advantage of TGS-mediated siRNA as a therapeutic means of targeting HPV infection is the innate ability to target numerable gene products if regulated by a common gene promoter and the heritable repressive epigenetic modifications that are passed to daughter cells, resulting in longer and more durable silencing effect (304).

Central to the uptake of RNAi by the general populous are cost and availability. RNAi poses unique challenges to both. The costs associated with the research, development, and implementation of an RNAi platform are considerable (308). Although the manufacturing capability of RNA and the necessary delivery platform needed to deliver the inherently unstable RNA into the host cell (309) is expanding rapidly in response to the uptake of mRNA vaccinations. These necessary investments translate to high treatment costs. FDA approved siRNA therapeutic yearly list costs include: patisiran (Onpattro), 451,430 USD; GIVLAARI (givosiran), 575,000 USD; and AMVUTTRA^®^ (Vutrisiran), 463,500 USD (310–312). These costs are considerable and would need to decrease in order to provide equitable access to RNAi-based therapeutics. Despite the initial costs, advancements in process design, delivery platform development, and RNA synthesis have enabled a steady decrease in the production costs associated with RNAi therapeutics, reducing costs and broadening the expected patient population (313). Significant to the promise of RNAi therapeutics is the unique adaptability to combat evolving viral pathogens and address several heritable genetic conditions (294, 309, 314). Following continued advancement in translational RNA research, improved access to RNAi therapeutics could enable widespread adoption and the subsequent treatment of numerable viral and genomic conditions currently without therapeutic options.

### 6.3 Delivery of siRNA or mRNA therapeutics and applications

Currently, siRNA delivery remains technically challenging on several fronts (315). RNAi delivery processes must prevent the destruction of the inherently unstable, naked RNA prior to entering the target cell and inducing silencing. Immune and metabolic factors dramatically limit delivery mechanisms as they must be able to overcome administrative, as well as vascular and cellular barriers (316). These challenges, therefore, require stabilizing modifications to siRNA sequences that can include 2′-O-methylation on the 3′ terminal ribose and/or carriers to facilitate the functional delivery of RNAi therapeutics (317). Three delivery carriers that serve to overcome these factors are nanoparticle delivery, GalNAc conjugates, and lentiviral vector delivery (318).

**Figure 4:**
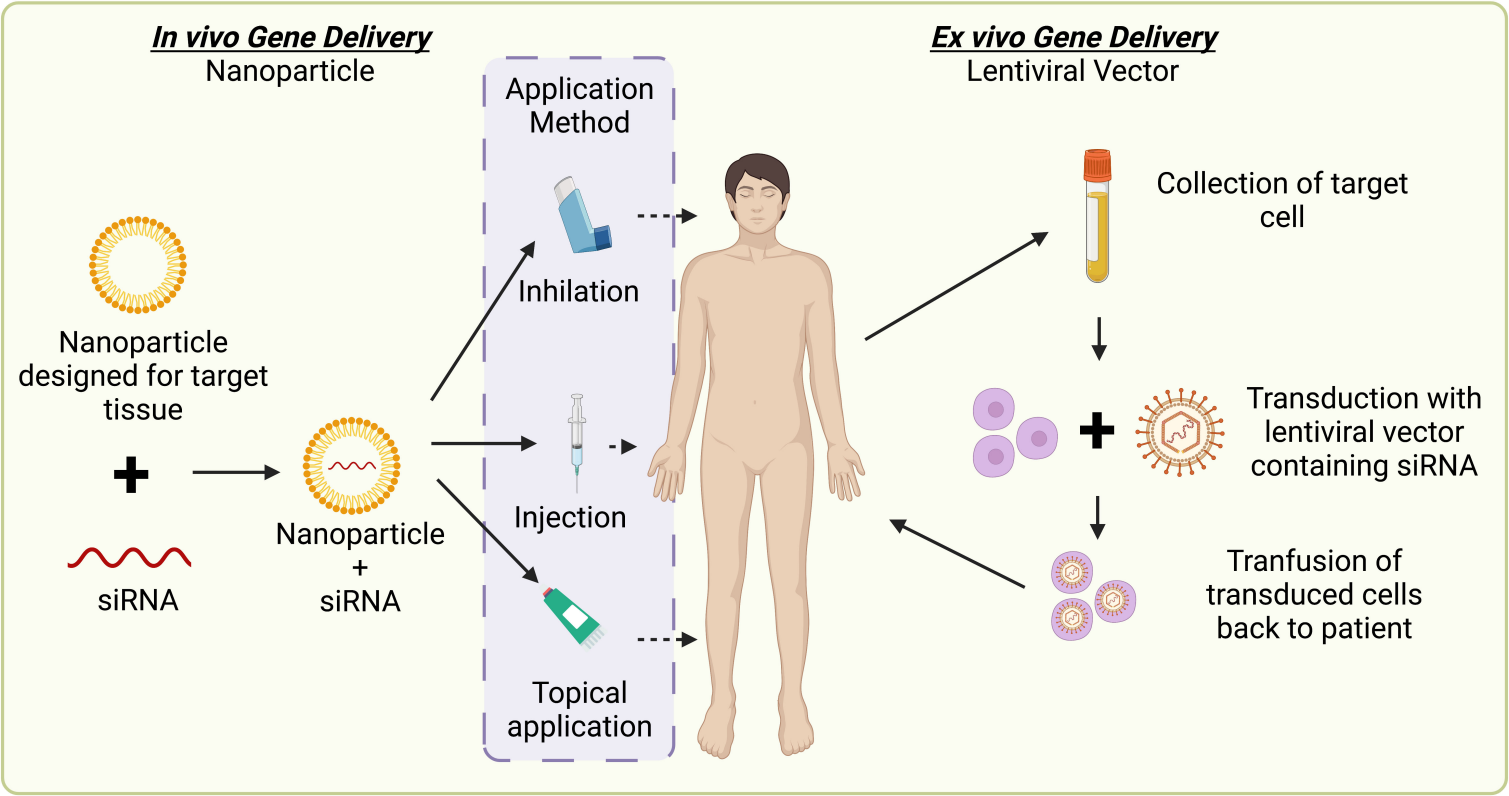
Comparative diagram of *in vivo / ex vivo* gene deliveries. *In-vivo* gene delivery via the use of nanoparticles. Conjugation of the nanoparticle designed for the target cell and the siRNA designed against the targeted genetic material are applied to the patient based of disease characteristics (i.e topical application for HPV infected cervical cells). *Ex-vivo* gene delivery via the use of lentiviral vectors requiring the collection of patient cells, transduction with the constructed lentiviral vector before re-introduction of the transduced patient cells back into the patient. Created with BioRender.com.

Delivery through the employment of chemically modified multifunctional nanoparticles serves to encapsulate the negatively charged naked siRNA and effectively facilitate transportation to the target cell without degradation caused by host barriers (**Figure 4**) (319) At the time of publication, one therapeutic utilizing nanoparticle technology: Onpattro (Patisiran) has been approved by the US FDA for therapeutic use *via* intravenous infusion (320). Additionally, recent success surrounding the safe and effective implementation of nanoparticle-delivered RNA-based vaccinations in response to SARS-Cov-2 heightens the potential for nanoparticle delivery of genetic-based therapies (276, 277).

GalNac-siRNA conjugates are another form of siRNA delivery that bind to the Asialoglycoprotein receptor widely present in liver hepatocytes facilitating siRNA entry. Four siRNA utilizing GalNAc conjugates: Oxlumo (Lumasiran), GIVLAARI (givosiran), Leqvio^®^ (inclisiran), and AMVUTTRA^®^ (Vutrisiran), have been approved by the United States FDA for therapeutic administration, however, the potential for GalNac-siRNA applications outside targeting liver disorders is less likely (321–325).

Lentivirus vectors expressing short hairpin (sh)RNA, which match the siRNA target sequence and include a linking loop between the sense and antisense strands, similarly allowing for the silencing of a targeted gene through the facilitated entry of targeted cells (326). The designed genetic material of the lentiviral genome can be transcribed to interfere with pathogenic viral RNA within the same cell, resulting in the downregulation of the targeted pathogenic viral protein (327). Lentiviral vector shRNA delivery uses an *ex vivo* gene therapy model, whereby patient cells are first isolated and transduced outside of the body prior to infusion back into the patient (**Figure 4**) (328). The use of lentiviral vector-mediated RNAi has been shown to reduce cellular proliferation and tumorigenesis of HPV-associated cancers and neuroblastoma *in vivo* and serves as a viable route for further inquiry (329, 330). However, there remains safety concerns with the lack of control of lentiviral integrations site into the host genome, and thus studies are ongoing to improve this aspect of lentiviral gene delivery (331).

## 7 Discussion

The epidemiological trends and biological mechanisms relating to HR-HPV and human carcinogenesis have been well documented. Additionally, there have been significant advancements made in preventative measures and therapeutic developments. Currently, the highest prevalence of HPV-related disease is in Sub-Saharan Africa, Eastern Europe, and Latin America (24, 25). Rates of cervical cancer are increasing and HPV+OPSCC are decreasing in western countries respectively. People living with immunodeficiencies are at a disproportionately high risk of persistent HPV infections and progressive disease compared to the general population.

Adequate immunosurveillance is crucial for viral elimination and preventing disease establishment. HPV-specific CD4^+^ and CD8^+^ T cells and inflammatory cytokines have been shown to negatively correlate with disease severity, with T cells being particularly important in targeting E6 and E7 epitopes (82). Where immunosurveillance is compromised, HPV disrupts critical signaling pathways such as JAK/STAT, apoptotic (TRAIL and XAF1), and IFN-γ (STAT1) resulting in immune evasion.

Lymphopenia is a common feature in those with specific primary and secondary immunodeficiencies at high risk of HPV-related disease. Patients with PIDs predisposed to HPV-related disease (103, 104), are predominantly associated with a CD4^+^ T cell lymphopenia, a decrease in CD8^+^ T cells and NK cells, and impaired TCR signaling; all factors important for targeting and eliminating HPV. CD4^+^ T cell lymphopenia is also evident up to 10 years post-renal transplant, a long-term effect associated with accelerated renal allograft decline and a high risk of cancer development compared to non-CD4^+^ T cell lymphopenic patients (154, 155). Despite ART improving prolonged increases in immune function (332) in PLWH, there is still a proportion who do not clear their lesions and are at high risk of developing HPV-driven cancer.

Tissue-resident memory T cell subsets and their roles in those with immunodeficiencies also require further investigation. This may be crucial to understanding tissue-specific responses in at-risk populations. Many reports focus on T cell responses in peripheral blood to assess immune response, which singularly is not the most accurate measure of immune function or cancer development. High CD8^+^ T_RM_ cell numbers have been correlated with better patient prognosis in HPV+OPSCC compared to their peripheral counterparts (243) and have shown to be a good prognostic indicator in many other malignancies including melanoma, ovarian and cervical cancer (333). There is a need to better understand tissue-specific responses to HPV, especially in immunocompromised individuals.

The clinical management of several malignancies remains consistent, irrespective of HPV status revealing a major limitation to current practices for poor responders and immunocompromised people. Currently, screening for HR-HPV detection or pre-cancerous lesions to aid in early diagnosis is only well established in cervical cancer compared to other HPV-driven cancers. While surgery can be combined with radiotherapy and chemotherapy, this approach is associated with substantial morbidity. In those established primary, secondary, and acquired immunodeficiency, opportunistic pathogens can establish disease. There is a need for more sophisticated, targeted treatments, especially for those with impaired immunity. Despite prophylactic vaccines showing high efficacy in the general population, long-term benefit in immunocompromised hosts is not well understood. Additionally, vaccine protection is limited to HR-HPVs naive to the host and therefore has major limitations as a preventative strategy. This is also applicable to HPV latency in infected cells. There is a need to address protection against LR-HPVs which can manifest severe clinical disease in immunodeficient individuals. There has been an acceleration in the investigation of cell-based therapies, immune checkpoint inhibitors, and therapeutic vaccines to address these limitations.

Host-directed therapies hold great promise for the treatment of patients with advanced-stage or relapsed HPV-associated cancers, however, there has been limited investigation into the efficacy of these in immunocompromised populations. Currently, PD-1/PD-L1 blockade has shown to improve overall survival in HPV+ individuals compared to chemotherapy, however, there is limited data on checkpoint inhibitor outcomes in specific at-risk populations. Furthermore, whether transplant patients and PLWH have different responses to checkpoint inhibition, regardless of HPV status, requires further elucidation. Several studies have shown that PD-1 inhibition (249) improves survival in HNSCC but has not been replicated in cervical cancer or ASCC, possibly suggesting divergent local tumor microenvironments at play (257, 259, 260). Additionally, the potential of therapeutic vaccines in combination with radiotherapy, chemotherapy or checkpoint inhibition requires further studies.

RNA-based therapeutic systems do not require an intact immune system (with the exception of mRNA vaccination) and hold great potential for the treatment of chronic pathological infections that resist traditional treatment approaches. RNA-associated downregulation of genes associated with HPV malignancies may inhibit viral genomic expression and reduce cellular proliferation by restoring normal intracellular p53 and pRb levels through the prevention or destruction of oncogenic RNA transcripts (334). Further development of RNA technologies against the early and late-stage promoters of the HPV genome could enable the silencing of multiple proteins simultaneously, reducing the likelihood of further cellular transformation. The disabling of viral replication and subsequent aberrant cellular development may facilitate the increased clearance of chronic HPV infection and result in improved clinical outcomes. Therefore, the investigation into RNA therapeutics serves as a logical route for next-generation antiviral therapeutics targeting HPV.

## 8 Conclusion

Given the inaccessibility of health infrastructure to provide sterilizing HPV vaccines in many parts of the world, and the existing high burden of HPV infection that can no longer be cured by prophylaxis, additional approaches are needed to treat the resulting pre-malignant and cancerous lesions. While there is a large body of research regarding the epidemiology of HPV-associated cancers in the immunocompromised population, it would be beneficial to investigate the effectiveness of currently available treatments in this sample patient population. There is a growing need to investigate the safety and immunogenicity of prophylactic HPV vaccines in PLWH. This will be important for determining the efficacy of these vaccines and the degree of risk of developing HPV-associated diseases. Although the use of ICB appears promising, particularly in regard to the use of PD-1 inhibitors for patients with HPV-driven cancers, treatment efficacy in immunocompromised individuals is not well understood. Given that people living with primary and secondary immunodeficiency have high rates of persistent, extensive, and malignant HPV-driven disease, such host-directed therapies may have divergent results from those seen in immunocompetent people. The rapid advancement of frontier gene-based RNA therapies, of which most do not require a functional immune system, may therefore play a pivotal role in eventually reducing the excess morbidity and mortality of HPV-driven disease in those most at-risk.

## Author contributions

RH and JA reviewed the literature, created, and designed tables and figures, and wrote the manuscript. SS and CA reviewed the literature and revised and proofread the manuscript. All authors contributed to the article and approved the submitted version.
